# A study on genetic aspects of male infertility in North-east Indian population, India

**DOI:** 10.1186/1753-6561-6-S6-P31

**Published:** 2012-10-01

**Authors:** Purnali Nath Barbhuiya, Anannya Gogoi, Giasuddin Ahmed, Rita Mahanta

**Affiliations:** 1 DBT-Sponsored Institutional Biotech Hubs, Cotton College, Guwahati, Assam-781001, India; 2; 3 Department of Biotechnology, Gauhati University, Guwahati, Assam-781014, India; 4 Department of Zoology, Cotton College, Guwahati, Assam-781001, India

## Background

Male infertility refers to the inability of a male to achieve a pregnancy in a fertile female [1]. Genetic factors are an important cause of male infertility. The present study was aimed to determine the role of Y-chromosome microdeletion and mitochondrial DNA (mtDNA) mutations - two major causes of male infertility, with special emphasis on patients of North-east India.

## Materials and methods

A total of 500 infertile male patients attending private infertility clinics of Guwahati, Assam, were selected for the study. Blood and semen samples were collected from the patients. Genomic DNA was isolated from both type of samples and PCR amplification was carried out using specific primer sets. The genes and sequence-tagged site (STS) markers included in the study are: DBY, USP9Y, PRY-2, RBMY, BPY-2, XKRY, CDY-1, CSPG4LY, DAZ, sY84, sY254, sY127, SY145, sY152 and sY153. All genes and STS were amplified efficiently in samples from 100 fertile men tested, but failed to be amplified in samples from fertile women. In order to study the role of mtDNA in sperm motility, semen DNA from 50 patients including 10 Asthenospermic, 20 Asthenoteratozoospermic and 20 oligoasthenoteratozoospermic patients and 20 fertile men were studied using specific primers for four mitochondrial genes, namely ND2, ND4, ATPase6 and ATPase8 followed by DNA sequencing.

## Results

Among the 500 patients included in this study, 130 (26%) were azoospermic, 185 (37 %) were oligozoospermic and 185 (37 %) were asthenozoospermic. Analysis of PCR amplified products showed that the frequency of Yq microdeletion in semen samples was 20.8% (104/500) but 18.6% (93/500) in blood samples, both of which lies in the range ( 0%-55%) as stated by Kihaile et.al [2]. In blood samples frequency of Yq microdeletion was as followed: AZFa 4% (20/500), AZFb 5.6% (28/500), AZFc 5.8% (29/500) and AZFd 14.6% (73/500). Similarly, in semen samples frequency of Yq microdeletion was as followed: AZFa 6.4% (32/500), AZFb 6.6% (33/500), AZFc 7.6% (38/500) and AZFd 18.4% (92/500). For mtDNA mutation study the mutations that were present in both fertile and infertile samples, were A4769G (M100M) (Silent) in ND2, A8701G (T59A) (Novel) and A8860G (T112A) (Novel) in ATPase6 gene. The mtDNA mutations which were found only in infertile patient group were given in Table 1.

**Table 1:** mtDNA mutations found in infertile patients

Type of Infertility	Gene	At nucleotide position	At amino acid position	Nature of mutation
Asthenozoospermia	ND2	T4823C	V118V	Silent
Asthenozoospermia	ND2	T4993C	L175F	Novel
Asthenoteratozoospermia	ND2	C4730T	T87T	Silent
Asthenoteratozoospermia	ND2	T5250G	L261V	Novel
Asthenoteratozoospermia	ATPase8	G8557C	L64F	Novel
Oligoasthenoteratozoospermia	ATPase6	T8614G	L30V	Novel
Oligoasthenoteratozoospermia	ATPase6	A8925G	T133T	Silent
Oligoasthenoteratozoospermia	ATPase6	G9064A	A180T	Novel
Oligoasthenoteratozoospermia	ND4	A10978G	L73L	Silent

## Conclusions

The study reveals that the frequency of Yq microdeletion is higher in semen samples than blood samples, possibly because most of the genes studied are testis-specific in nature. This is in accordance with the previous studies [3,4]. In both types of samples AZFd regions has highest frequency of Yq microdeletion. The mtDNA mutations common in fertile and infertile patients have also been reported by other Indian researchers [5], but doubted to play any role in infertily [6]. The mutation G9064A in ATPase6 has also been reported to play a role in female infertility [7]. Beside these, the other mtDNA mutations observed in the present study have not been reported previously.
